# What Can Determine the Length of an Open Nonendoscopic Thyroidectomy Incision?

**DOI:** 10.1055/s-0036-1584169

**Published:** 2016-05-10

**Authors:** Nilesh R. Vasan, Benjamin Collins

**Affiliations:** 1 Department of Otorhinolaryngology–Head and Neck Surgery, The University of Oklahoma Health Sciences Center, Oklahoma City, Oklahoma

**Keywords:** head and neck surgery, endocrine surgery, thyroid gland, advocacy

## Abstract

**Objectives** Surgeons are now utilizing small incisions when performing thyroidectomy. This study evaluated the association between patient weight, nodule size, and maximum thyroid diameter and the length of an open thyroidectomy incision.

**Study Design** Retrospective analysis of 32 consecutive patients.

**Subjects and Methods** Patient demographics, clinical exam, ultrasound findings, operative findings, and pathology were recorded.

**Results** Of the 32 patients (81% women), 27 underwent a hemithyroidectomy. The mean patient weight was 194 lbs. The mean clinical nodule diameter was 3.46 cm, and the mean maximum thyroid diameter was 5.91 cm. The mean incision size was 5.13 cm. Independently, patient weight, maximum thyroid diameter, and maximum nodule diameter were shown in regression models to be statistically significant predictors of incision size. In stepwise regression analysis that included all three listed variables, maximum thyroid diameter was the most significant predictor of incision size ( *p* < 0.0001).

**Conclusions** Surgeons may determine the length of the incision using clinical and radiologic parameters, but most probably use their subconscious clinical judgment and the challenge of utilizing a very small incision for this operation. This study has shown that maximum thyroid diameter is the most significant determinant for the incision but that nodule size and patient weight are also significant factors. This study is evidence-based medicine level III.

Thyroidectomy is a common operation performed by several disciplines. The usual technique requires a midline transverse anterior cervical incision to access the thyroid gland. The length of the incision varies depending on the thyroid lesion and the patient's habitus, as well as the surgeon's comfort level. As patients' expectations regarding aesthetic appearance following surgical procedures have increased, surgeons have begun to dramatically reduce the length of the thyroidectomy incision to minimize the scar.

To improve cosmesis, minimally invasive video-assisted thyroidectomy was first described by Miccoli and colleagues. 1 2 With this specialized endoscopic technique, an incision length of 1.5 cm was used to remove nodules less than 35 mm in largest diameter in a thyroid gland that was less than 20 mL in volume as determined by ultrasound. 2 Obviously, only a small percentage of patients with very small nodules can be treated in this manner.

Robotic thyroidectomy to avoid an anterior neck scar is popular in Asia but has not been widely practiced within North America, and open approaches remain commonplace.

This study evaluated the association between patient weight, nodule size, maximum thyroid diameter, and incision length of an open non-video-assisted thyroidectomy for 32 consecutive patients.

## Methods

From August 2004 to May 2008, data from a cohort of 32 consecutive patients treated by a single surgeon managed at three separate private practice institutions was retrospectively collected and analyzed. The surgeon is a fellowship-trained high-volume head and neck surgeon. During this period of private practice, he sustained a moderate volume of thyroidectomies. Patients requiring revision procedures or neck dissection were excluded. The patient demographics, clinical examination, ultrasound findings, operative findings, and pathology were recorded. Indirect laryngoscopy was performed both pre- and postoperatively to assess vocal cord movement. All incisions were measured preoperatively.

The preoperative diameter of the thyroid and/or nodule was measured clinically within the office with ultrasound. A fine needle aspirate (FNA) or ultrasound-guided FNA was commonly performed.

**Fig. 1:**
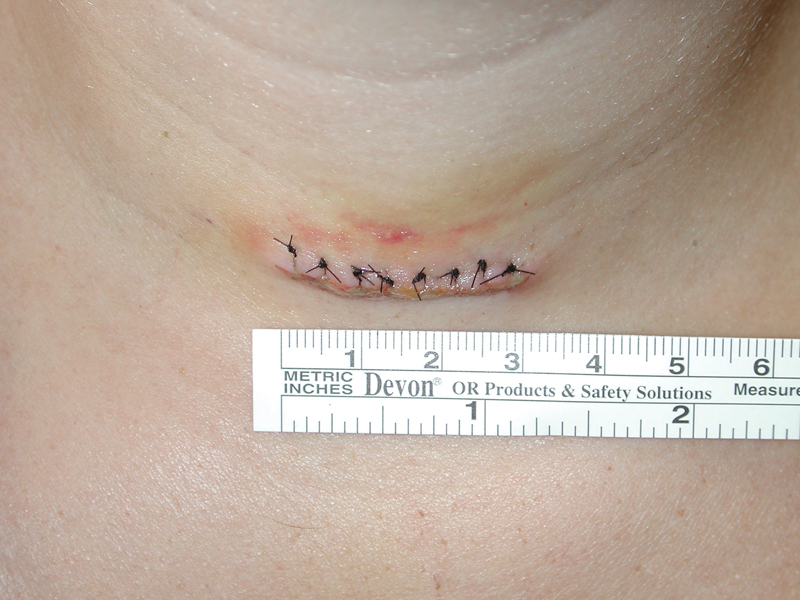
Length of incision in centimeters.

The technique required the patient's neck to be extended with a shoulder roll. The transverse incision was illustrated with a pen and measured. The incision length was individualized for each patient with some incisions planned smaller than others. This decision often took into account many factors including size of the neck, size of the thyroid nodule, and subconscious factors at the time of the operation. Usually, the incision was marked an extra 5 to 10 mm on either side of the prospective incision in case the wound required extension. Great care was taken either to incorporate the incision within a natural skin crease or to make the incision parallel to other skin creases within the anterior neck. In patients with no skin creases, the incision was illustrated in the preoperative area while the patient was awake. All incisions were within the midline and symmetric. Because the recurrent laryngeal nerve enters the larynx at the level just above the cricoid cartilage, for nerve preservation the smaller the incision, the higher it should be. To help facilitate a smaller incision, the incisions were placed slightly higher on the neck between the cricoid cartilage and sternal notch ( Fig. 1 ). Lower incisions in general should be larger. All patients had the skin infiltrated with 1% xylocaine with 1:100,000 epinephrine, and the incision was made with a scalpel.

The subplatysmal flaps were elevated both superiorly and inferiorly with division of the midline cervical fascia to allow access to the thyroid gland. In the majority of cases, the strap muscles were not divided unless the patient had a very large goiter. The superior and inferior thyroid vessels were usually secured with ties.

After the superior and inferior thyroid poles were mobilized, the lobe was retracted medially. The recurrent laryngeal nerve was visually identified as well as the parathyroid glands, which were preserved. The ligament of Berry was divided, and the thyroid was then redirected off the trachea with the isthmus. The recurrent laryngeal intraoperative nerve monitor was not used in this series of patients. Ideally, having two assistants helps expedite this procedure with retraction; however, this operation can be easily undertaken with one assistant. If access and safe removal was difficult with the planned incision length, it was extended in some instances by 0.5 to 1 cm on each side of the midline. The incision length recorded for the study is the final total length and accounts for any extension.

As this study was performed in three different institutions (Hospital of Saint Raphael, Midstate Hospital, and Middlesex Hospital, Connecticut), there were some minor variations in gross histologic assessment. In the majority of cases, the entire thyroid gland or hemithyroidectomy and isthmus were measured and weighed. In three cases, however, the total dimensions were not available because the pathology staff had divided the lobes and isthmus first and given these dimensions separately. In these cases, the smallest dimension from each portion of the gland was added and recorded. With this method, a highly conservative and likely smaller total measurement was obtained for these patients.

Statistical analysis was performed using SAS v. 8.2 (SAS Institute, Cary, North Carolina, United States). Numerical data is presented with means ± standard deviations and minimum and maximum values. Simple linear and multiple stepwise regression analyses were performed to investigate the association between the various independent parameters and the outcome of interest (incision size).

## Results

Thirty-two consecutive patients who met the inclusion criteria were assessed, of whom 26 (81%) were women. The mean age of these patients was 47 (range 25 to 83) years. Twenty-seven (84%) patients underwent a hemithyroidectomy and isthmusectomy. One patient had papillary thyroid cancer and 7 (22%) required operation for compressive symptoms. Incidental nodules were found in 14 (44%) patients, who then required a thyroid operation. Twenty-seven (84%) patients had a neck mass.

The majority (30/32; 94%) of these patients underwent FNA as part of their initial workup. Nine (28%) of these patients did not have FNA within the office as the nodules were not palpable. Of these patients, 7 (78%) required ultrasound-guided FNA. Cytopathology indicated benign cells in 12 (40%) patients, indeterminate in 5 (17%), and suspicious cells in 13 (43%).

Frozen sections were performed on 27 (84%) patients. Of the 5 patients who required a total thyroidectomy, in only 1 case was the specimen sent for frozen section. Frozen section did not reveal carcinoma in any of these patients, with 23 (85%) demonstrating benign cells and 4 patients having “intermediate” cytology. Subsequent permanent histology confirmed benign disease in 97% of these patients.

The mean weight was 194 (range 130 to 400) pounds, and the mean clinical nodule diameter was 3.46 cm (range 0.9 to 7cm). Pathology specimens had a mean weight of 45.4 g (range 2 to 267) and a mean pathologic nodule diameter of 2.99 cm (range 0.6 to 6.3 cm). The mean maximum thyroid diameter of the specimens was 5.9 cm (range 2.7 to 14cm).

The mean incision length was 5.13 cm (range 3 to 10 cm), with 47% (15/32) of patients in this series having either a hemithyroidectomy/isthmusectomy or total thyroidectomy with an incision length of 4 cm or less. Only one patient exhibited a change in voice pitch postoperatively with normal vocal cord movement. There were no other complications.

An analysis was performed to correlate the mean incision size with patient weight ( Table 1 ), clinical nodule size ( Table 2 ), pathologic nodule diameter ( Table 3 ), and maximum thyroid diameter ( Table 4 ). The majority of patients weighed between 100 and 220 pounds (75%), whose mean incision length was 4.83 cm ( Table 1 ). The mean incision lengths were found to be ∼1 to 3 cm longer than the thyroid nodule maximum diameter both clinically as well as on gross pathology ( Tables 2 and 3 ).

**Table 1:** Mean incision size by patient weight

Weight (pounds)	*n*	Mean	SD	Minimum	Maximum
Value not available	1	3.00		3.00	3.00
100–220	24	4.83	1.46	3.00	8.00
220–300	5	5.80	0.44	5.00	6.00
>300	2	8.00	2.83	6.00	10.00

Abbreviation: SD, standard deviation.

**Table 2:** Mean incision size by clinical thyroid nodule diameter

Clinical maximum nodule diameter (cm)	*n*	Mean	SD	Minimum	Maximum
Value not available	2	7.00	1.41	6.00	8.00
≤2.0	6	3.50	0.55	3.00	4.00
2.0–4.0	13	4.85	0.90	4.00	6.00
>4.0	11	6.00	1.95	4.00	10.00

Abbreviation: SD, standard deviation.

**Table 3:** Mean incision size by pathologic thyroid nodule diameter

Pathologic maximum nodule diameter (cm)	*n*	Mean	SD	Minimum	Maximum
Value not available	2	7.00	1.41	6.00	8.00
≤2.0	9	3.78	0.67	3.00	5.00
2.0–4.0	14	4.93	0.92	4.00	6.00
>4.0	7	6.71	2.06	4.00	10.00

Abbreviation: SD, standard deviation.

**Table 4:** Mean incision size by pathologic maximum thyroid diameter

Pathologic maximum thyroid diameter (cm)	*n*	Mean	SD	Minimum	Maximum
≤4.0	9	3.78	0.44	3.00	4.00
4.0–8.0	17	4.94	0.97	3.00	6.00
8.0–10	3	6.67	1.15	6.00	8.00
>10.0	3	8.67	1.15	8.00	10.00

Abbreviation: SD, standard deviation.

The analysis of mean incision length in relation to the maximum thyroid diameter on gross pathology, however, revealed that the mean length of the incision was either similar to the maximum thyroid diameter or smaller ( Table 4 ).

Linear regression analyses were performed for patient weight, nodule diameter (pathologic), and maximum thyroid diameter in relation to incision size. All three variables—patient weight ( *p* = 0.0014), nodule diameter ( *p* < 0.001), and thyroid diameter ( *p* < 0.001)—were individually significant predictors of incision size ( Table 5 ).

**Table 5:** Linear regression analysis

Outcome	Independent variable	Regression equation	*p* value
Incision size	Patient weight	Incision size = 2.44 + 0.014 × patient weight	<0.0001
Incision size	Pathology maximum thyroid diameter	Incision size = 2.15 + 0.50 × thyroid maximum diameter	<0.0001
Incision size	Pathology nodule diameter	Incision size = 2.40 + 0.75 × nodule maximum diameter	<0.0001

**Fig. 2:**
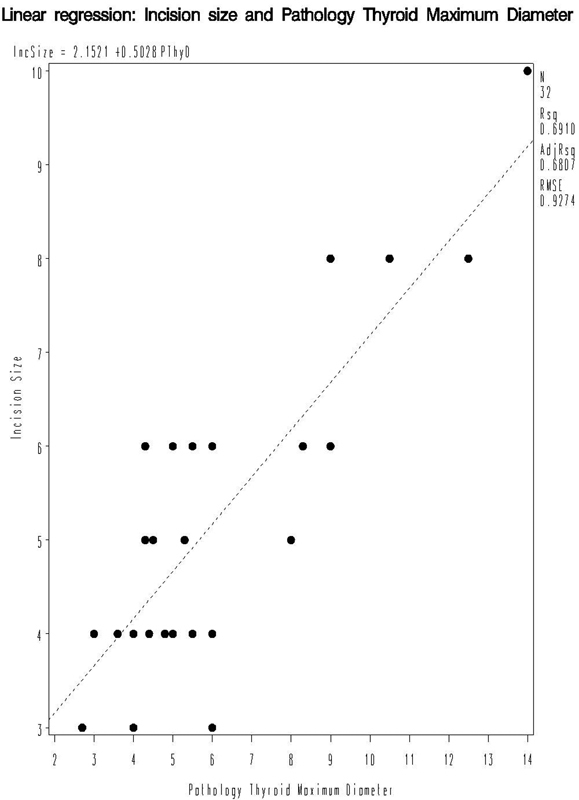
Linear regression: incision size and pathology thyroid maximum diameter.

A diagrammatic representation and regression equation for maximum thyroid diameter are shown in Fig. 2 . Similar equations and graphs can be constructed for the other variables that were analyzed. Using the regression equation, one can predict the incision size based on these variables, provided the value of the given parameter is within the range of values recorded in this study.

A multiple regression analysis found that pathologic maximum thyroid diameter and patient weight were the most significant determinants of incision size ( *p* < 0.0001 and *p* < 0.03, respectively). Nodule diameter was not a significant predictor in the presence of the other two variables. However, as mentioned previously, individually all three variables were statistically significant in predicting incision size and could therefore be used to accurately predict the incision size for a thyroidectomy operation.

## Discussion

With the advent of endoscopic techniques and new technologies such as the harmonic scalpel, surgeons are now able to perform complex procedures without the need for a large incision. Within the head and neck area, video-assisted thyroidectomy has allowed surgeons to remove either one or both lobes of the thyroid using only a 1.5- to 2-cm incision. This technique, however, requires specialized equipment to perform the operation, has a steep learning curve, needs extra staff, and can only be utilized in specialized high-volume units. Unfortunately, this endoscopic technique can only be used in 10 to 15% of thyroid patients, with the majority of patients still needing a standard open procedure. 3

This study has shown in a small series of patients with a wide range of thyroid dimensions that smaller incisions can be used to adequately perform the operation compared with a Kocher incision. Forty-seven percent of incisions were 4 cm or less, with a mean incision size of 5.125 cm for all patients.

Terris et al compared conventional thyroidectomy (group A, 6- to 12-cm incision), minimally invasive nonendoscopic thyroidectomy (group B, 3- to 6-cm incision), and video-assisted (endoscopic) thyroidectomy (group C, 1.5- to 2-cm incision) in a prospective nonrandomized study. 4 The mean incision lengths for groups A, B, and C were 9.24, 4.64, and 2.43 cm, respectively. The surgical approach was selected on clinical judgment, nodule size, thyroid gland size, and degree of obesity. No measurements were given for these variables in this study, but the mean incision length in group B was slightly smaller than in this series.

Ultrasound is frequently used to determine the size of the thyroid nodule and the thyroid gland itself. Some authors require a total thyroid volume of 20 to 30 mL or less before advocating a video-assisted technique. 2 5 In this series, our ultrasonographers did not document thyroid volume but instead measured the nodule and lobe in three dimensions. This simple method of thyroid measurement may allow the surgeon to better plan the incision length compared with an estimated volume recording of the thyroid gland.

The limitations of this study include its small sample size and a single surgeon's experience. The factors of maximum thyroid diameter, nodule size, and patient weight are significant predictors of incision size in this study. In addition to the factors found in this study, other factors like patient height, skin thickness, height of thyroid, and retrosternal extension may also potentially affect the size of the incision.

The benefits of a smaller thyroid incision cannot be disputed. Other than an aesthetically pleasing scar, patients have been shown to suffer less pain compared with longer incisions with no difference in complication rate. 2 5 6 Minimally invasive nonendoscopic techniques are more easily achieved and reproduced for the majority of surgeons with less access to sophisticated equipment and less staff than those able to perform endoscopic approaches. Postoperative care of the scar, such as avoidance of direct sunlight on the incision, application of moisturizing creams, meticulous wound cleaning, and application of Silastic (Dow Corning, Auburn, Michigan) (or similar) dressings, also contributes to a well-healed and aesthetically pleasing incision, and this care should also be discussed with the patient.

## Conclusion

The use of a smaller incision for thyroidectomy is becoming increasingly common. Some surgeons may determine the length of the incision using clinical and radiologic parameters, but most probably use their subconscious clinical judgment and the challenge of utilizing a very small incision for this operation. This study has shown that maximum thyroid diameter is the most significant determinant for the incision, but nodule size and patient weight are also significant factors.
